# Personalising renal function monitoring and interventions in people living with heart failure: a protocol for co-designing a care pathway in the RENAL-HF programme

**DOI:** 10.3399/BJGPO.2025.0099

**Published:** 2026-02-11

**Authors:** Suzy C Hargreaves, Christopher J Armitage, Benjamin C Brown, Dawn Dowding, Jennifer Downing, Mark Goodall, Alison Gummery, Carolyn Lees, Emma Sowden, Nefyn H Williams, Bridget Young, Munir Pirmohamed, The RENAL-HF Consortium

**Affiliations:** 1 Department of Primary Care and Mental Health, Institute of Population Health, University of Liverpool, Liverpool, United Kingdom; 2 Manchester Centre for Health Psychology. University of Manchester and NIHR Greater Manchester, Patient Safety Research Collaboration, University of Manchester, Manchester, United Kingdom; 3 School of Health Sciences, University of Manchester, Manchester, United Kingdom; 4 Department of Pharmacology and Therapeutics, Institute of Systems, Molecular & Integrative Biology, University of Liverpool, Liverpool, United Kingdom; 5 School of Allied Health Professions and Nursing, Institute of Population Health, University of Liverpool, Liverpool, United Kingdom; 6 Department of Public Health, Policy and Systems, Institute of Population Health, University of Liverpool, Liverpool, United Kingdom

**Keywords:** renal function, optimising care, heart failure, general practitioners, primary healthcare

## Abstract

**Background:**

Heart failure affects almost one million people in the UK and is increasing in prevalence. Many drugs used to treat heart failure impair renal function and can lead to hospitalisation. Adverse drug problems can be partially mitigated through regular renal monitoring and optimising of drug dose and choice to prevent deterioration of kidney function. This protocol describes part of a wider research programme: personalising renal function monitoring and interventions in people living with heart failure (RENAL-HF).

**Aim:**

The aim of RENAL-HF is to develop improved processes in primary care to manage kidney health in people living with heart failure.

**Method:**

The protocol covers gathering views of healthcare professionals, patients, and carers, to co-develop a care pathway for use in primary care. Using a mixed-methods approach, the work comprises the following six stages: (1) understanding current practice of optimising heart failure treatment while preserving renal function; (2) co-designing a care pathway including personalised renal function monitoring, thresholds for intervention and clinical guidelines; (3) decision making to identify elements that will support the care pathway; (4) developing training materials for primary care to enable use of the care pathway; (5) testing the usability of the prototype care pathway; and 6) a feasibility and acceptability study to inform the pre-clinical development and usability of the care pathway ahead of a cluster randomised control trial (RCT).

**Conclusion:**

All stages will elicit evidence from primary care practices, practitioners, and patients with which to assess and refine the care pathway. The evidence will inform how algorithm-guided individualised treatment can be implemented to improve the outcomes of patients with heart failure.

## How this fits in

Many drugs used to treat heart failure impair renal function. There is some guidance on monitoring renal function in people with heart failure following changes in medication, but this does not consider individuals’ clinical factors, and there is a marked variation in clinical practice. This protocol describes a work package that forms part of a wider programme of research (personalising renal function monitoring and interventions in people living with heart failure [RENAL-HF]). RENAL-HF involves various integrated studies to develop better processes in primary care to manage kidney health in people with heart failure, through the creation of a standardised care pathway based on individual patient data, predicted by an algorithm. This protocol article describes the understanding of current practice around the optimisation of heart failure treatment while preserving renal function; the co-creation of a care pathway; and a feasibility and acceptability study, ahead of a future cluster randomised control trial (RCT).

## Introduction

Heart failure is a condition where the heart is unable to pump blood effectively to meet the body’s oxygen needs.^1^ It affects almost one million people in the UK and its prevalence is increasing.^2,3^ Despite modern treatment reducing morbidity and improving survival for this population, heart failure is a common contributing factor to acute and chronic kidney disease.^4^ Renal impairment associated with heart failure drugs is the second most common adverse drug reaction resulting in hospitalisation,^4,5^ meaning even a small decline in renal function may precipitate costly hospital admission putting further pressure on the healthcare system.^6^

Clear guidance on the timing and frequency of reviews is lacking.^4,7^ There is a need to standardise care processes for this population to reduce variability in clinical practice and improve outcomes by developing an improved care pathway within primary care. It is recommended that care processes are standardised for patients who are at most risk of declining renal function, as such standardisation would improve patient identification and health outcomes, and reduce hospital admissions.^4^

### Overview of the wider programme (RENAL-HF)

**Figure 1.**
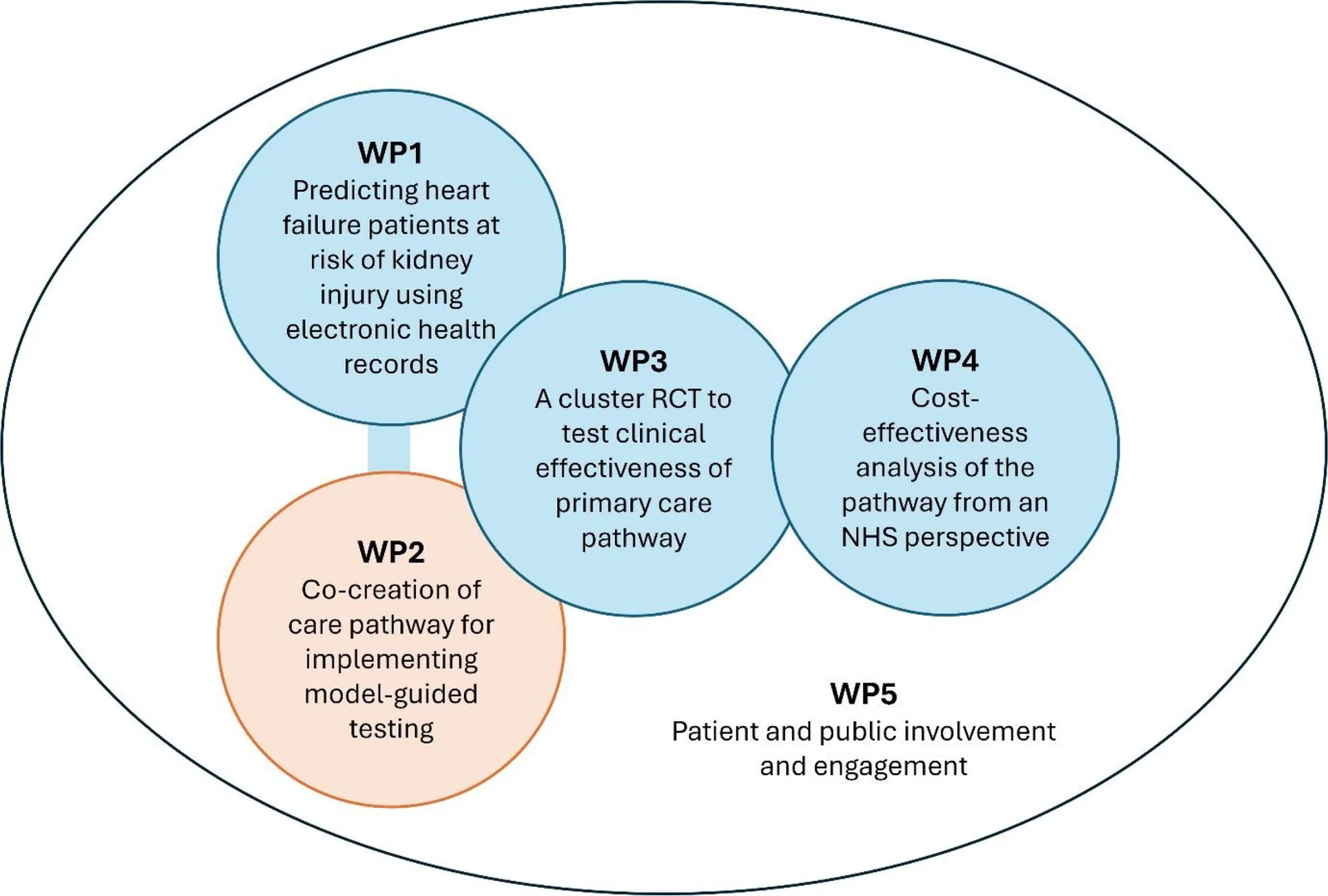
Co-creation of the care pathway (WP2) within context of wider RENAL-HF programme. RCT = randomised controlled trial. WP = work package

**Figure 2.**
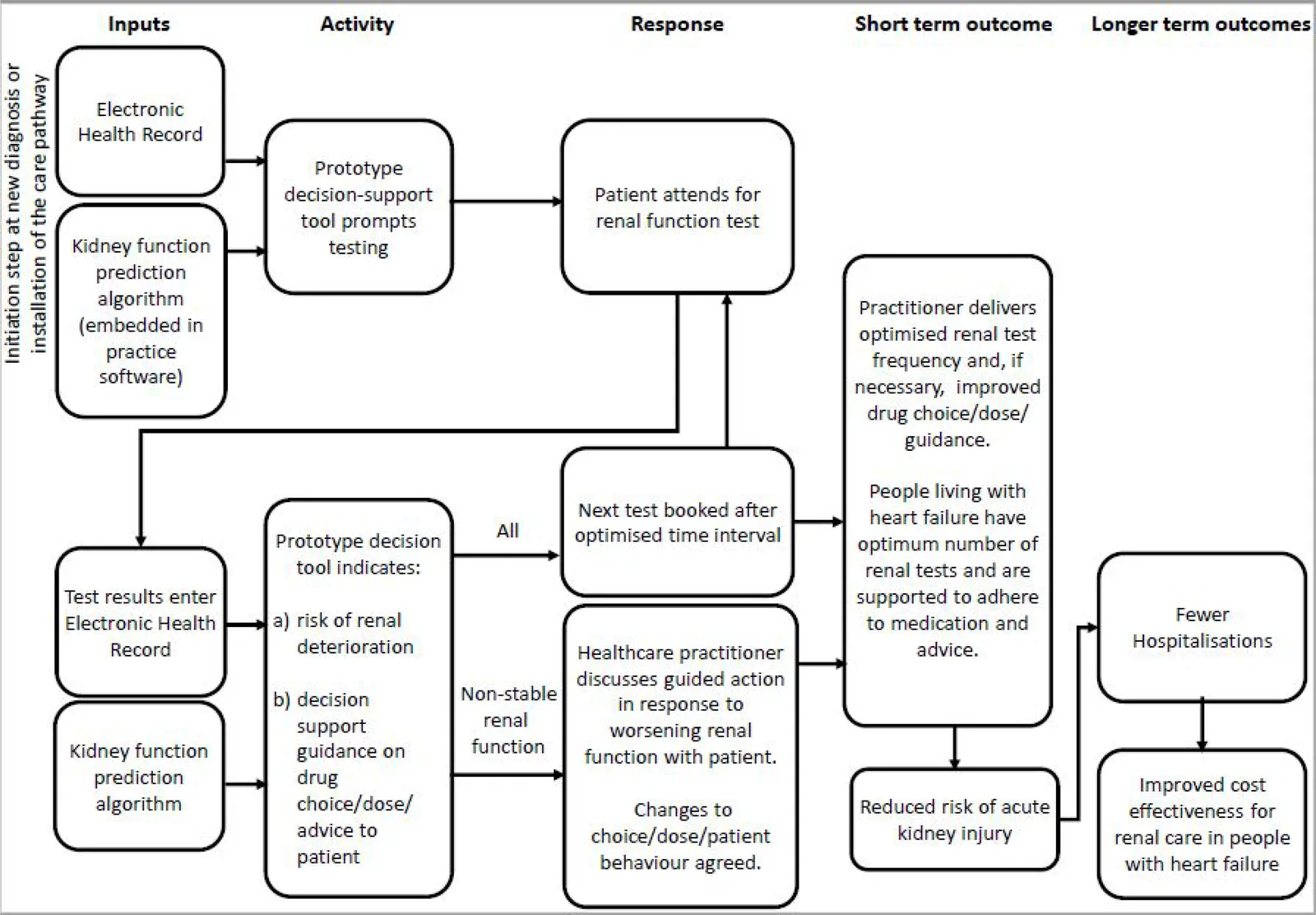
Logic model describing the RENAL-HF programme and care pathway

The National Institute for Health and Care Research (NIHR) Programme Grant for Applied Research (ISRCTN75284771),^8^ personalising **renal** function monitoring and interventions in people living with **h**eart **f**ailure (RENAL-HF) consists of five interconnected work packages (WPs). The study described in this protocol forms a work package (WP2) (comprising stages 1–6) in this wider programme of research. Figure 1 shows an overview of RENAL-HF and the five WPs, and Figure 2 shows the overall logic model for the research programme, including inputs, activity, response, and short and longer-term outcomes.

The overall aim of RENAL-HF is to develop better processes in primary care to manage kidney health in people with heart failure. We will use medical records to (a) develop technology to predict how often each person with heart failure needs a renal function blood test and (b) inform the development of expert advice for healthcare practitioners on how best to adjust medication. In WP1 we will use advanced analytical methods and electronic healthcare records to refine the accuracy of an algorithm that predicts the change in renal function in patients with heart failure. WP3 comprises an RCT to test clinical effectiveness of the pathway developed in WPs 1 and 2. We will determine if this approach is more effective or better value for money than the current standard of care by conducting a cost-effectiveness analysis of the pathway (WP4). WP5 will develop and manage patient and public involvement and will integrate public advisers throughout the whole programme to ensure the patient voice is present at all stages of the work.

Working with patients, primary care practitioners, and specialists (including cardiologists and nephrologists), we will find the best way of implementing this personalised approach to renal function monitoring and interventions through the co-design of a care pathway.

## Method

**Figure 3.**
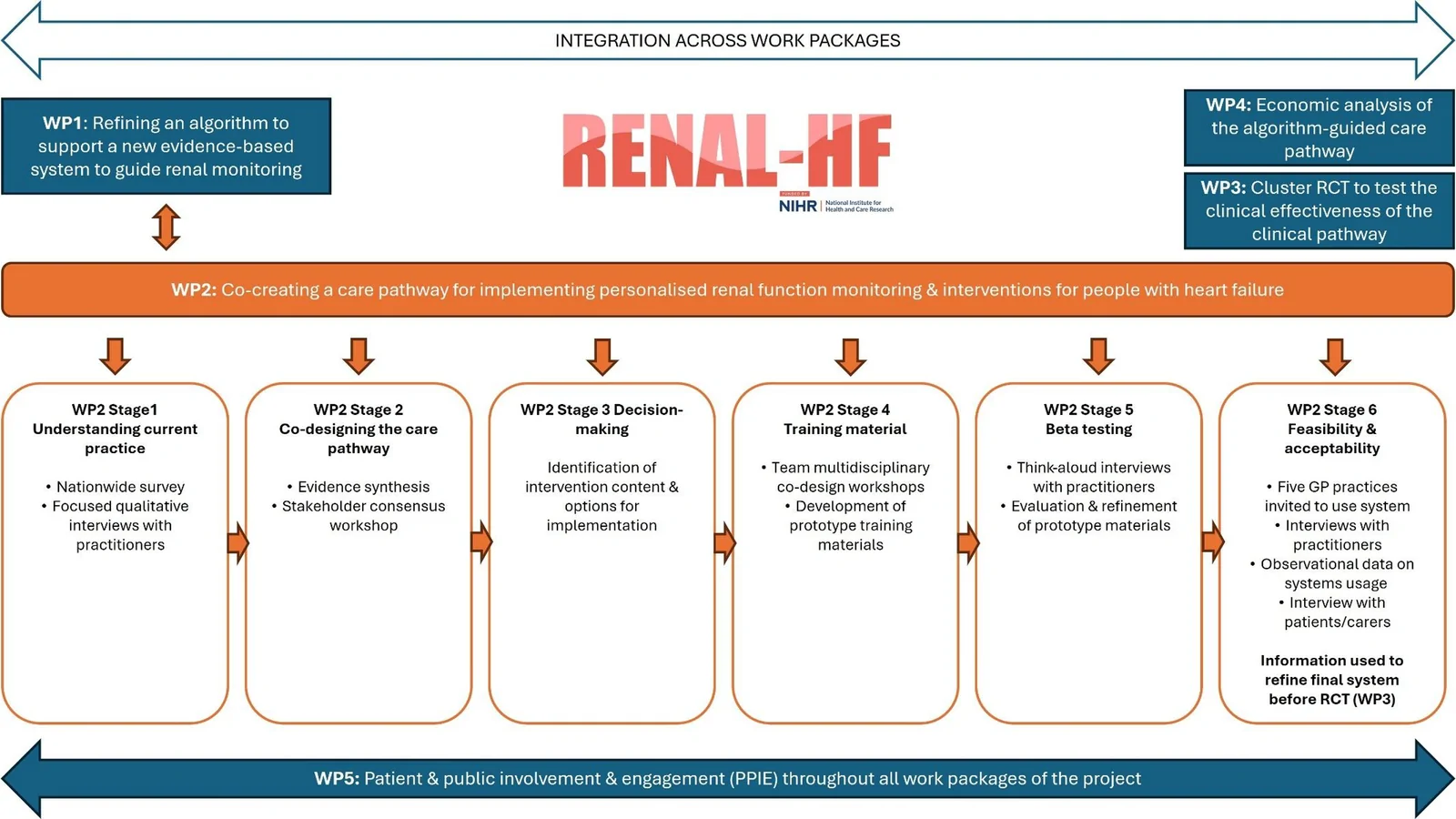
Stages 1–6 of WP2 within context of wider RENAL-HF programme. RCT = randomised controlled trial. WP = work package

WP2 will focus on the co-creation of a care pathway, using mixed methods, with six stages (see Figure 3). All quantitative analyses will be a mix of descriptive and more in-depth statistical models, such as multi-variable regression to predict behaviour change. Microsoft Excel and IBM SPSS Statistics (version 29.0.2.0) will be used to analyse the data. Qualitative analyses will be underpinned by the behaviour change wheel (BCW) theory.^9,10^ Following inductive and reflexive thematic analysis and framework analysis^11,12^ using NVivo (version 14), data will be deductively mapped onto the Capability, Opportunity, Motivation, and Behaviour model (COM-B) and the Theoretical Domains Framework (TDF).^9,10,13^ The methods for each stage are outlined below.

### Understanding current practice

Stage 1 aims to understand current practice, and establish patient and practitioner views on standards of care through a survey of healthcare professionals in England, including GPs, pharmacists, and nurses. This will be co-developed by the research team and patient and public involvement and engagement (PPIE) and delivered through YouGov. A sample of 600 participants; 115 GPs, 115 pharmacists, and 370 nurses will be recruited. Data will be analysed quantitively. Open-ended questions will be thematically analysed and mapped onto the TDF.^9,10,13^ Furthermore, this stage aims to identify barriers and facilitators to optimising the treatment of patients with heart failure while preserving renal function. This will be achieved by conducting brief focused qualitative interviews with GPs, nurses, and pharmacists recruited from primary care practices to generate options for intervention functions. The interview guide will be informed by the BCW^9,10^ and will be used to understand drivers of optimising treatment of people living with heart failure while preserving renal function. There will be a sample of up to 51 participants. Analysis includes inductive thematic coding and mapping onto the TDF.^13^

### Co-designing the care pathway

Researchers will work closely with stakeholders, the WP1 team, and an expert clinical panel (comprised of cardiologists, nephrologists, clinical pharmacologists, and GPs) to develop prototype training material to ensure optimal design and uptake of the RENAL-HF care pathway. This will include personalised renal function monitoring, thresholds for intervention, and clinical guidelines.

The material generated in WP1 and outputs from WP2 stage 1 will be synthesised during a series of meetings to generate possible elements of the care pathway. Proposals will be rated in stakeholder workshops. The clinical expert panel will identify clinical parameters for key outputs and treatment changes that may be required based on renal function deterioration. Stakeholder consensus workshops will be held to ensure views of key stakeholders are included in design of the intervention. Five stakeholder groups of nine individuals each, including patients, pharmacists, nurses, GPs, and other key informants, will engage in three rounds, rating the proposals created from the synthesis of stage 1 and of WP1. The RAND/UCLA Appropriateness Methods (RAM) will be used^14,15^ to reach group consensus.

### Decision making

Elements that will support optimal implementation of the evidence-based system created in stage 2 will be considered in stage 3. The BCW will be used to identify intervention functions and behaviour change techniques that will be most likely to achieve the change required.^10^ Evaluation of the intervention will be conducted according to the acceptability, practicability, effectiveness or cost-effectiveness, affordability, and safety or side-effects (APPEASE) framework defined in the BCW.^10^

### Training materials

Prototype training material will be developed for primary care teams (GPs, pharmacists, nurses, and care navigators) and refined through beta testing (stage 5) and a feasibility and acceptability study (stage 6), ready for the cluster RCT in WP3. Co-design of the behaviour change interventions will be completed in a series of up to three workshops made up of the wider RENAL-HF research team. Findings in stages 1–3 and WP1 will help determine the type of content to be included in training materials. Materials will include rationale for the study, how the algorithm and interventions were developed, evidence bases for undertaking interventions (increased renal function monitoring, changing drugs or doses, and so on), how to use the care pathway, and how it is being used. The format for the materials will be informed by stakeholders and will include videos hosted on a dedicated website and integrated self-guided learning materials.

### Beta testing

Evaluation of the usability of the prototype materials (care pathway and clinical guidance tool) developed in previous stages will be conducted through a series of rapid rounds of beta testing. Evaluation will use ‘think-aloud’ interviews with 36 primary care staff (nine from each sub-group; GPs, pharmacists, nurses, and care navigators). This will provide insight into how primary care staff interact with the systems and refinements will be made where appropriate following this evaluation.^16^ During the interviews, participants will be instructed to verbalise their thoughts while conducting predefined tasks with the care pathway within a dummy environment. The prototype training materials will be refined according to the findings of this stage in preparation for the feasibility and acceptability study. Data will be analysed using thematic analysis.^11^

### Feasibility and acceptability sub-study

Stages 1–5 will inform the pre-clinical development and usability testing of an algorithm-guided care pathway to improve the kidney health of people with heart failure. Stage 6, a feasibility and acceptability study of the whole process in a ‘real-world environment’, will be conducted to inform final refinements before the start of the cluster RCT (WP3). The algorithm will be installed in five GP practices and will be used for a minimum of 6 weeks. Quantitative data will be gathered on the number of patients with heart failure, the number of patients with heart failure who already have renal impairment, and those who develop renal impairment.

Patients will be identified by the RENAL-HF care pathway and analytics will be used to understand the number of times the pathway is used; by whom (for example, job role); and in what circumstance. We will assess the number of alerts triggered and ignored; actions taken (for example, patient contacted for blood test performed in accordance with, and/or contrary to the suggested schedule); and changes in treatment after using the pathway. Data on referrals to cardiology and renal services in secondary care and emergency hospital admissions will also be collected. To assess the health inequalities impact of this intervention, data related to sex, ethnicity, age, and postcodes for patients and the general practice will be collected. This information will contribute to a health inequalities impact assessment informed by the Health Inequalities Assessment Toolkit (HIAT).^17^

Qualitative evaluation will be undertaken in the following two parts:

1. In-depth semi-structured interviews will be conducted with a purposive sample of primary care practitioners from each of the five participating practices. Interview guides will be informed by the BCW and the COM-B model to explore facilitators of and barriers to using the pathway. We will recruit a purposive sample of up to 34 practitioners, including those who did and did not engage with the care pathway. Data analysis will use thematic coding and mapping on to the TDF^13^ and will be informed by the HIAT.^17^
2. In-depth, semi-structured interviews with a purposive sample of up to 25 people living with heart failure and their informal carers will take place to examine the acceptability and health inequalities impact of the care pathway from the patient perspective. The sampling will take place across the participating practices, aiming for a balanced coverage in terms of patient sociodemographics (sex, ethnicity, and socioeconomic status); those with normal renal function; and those with chronic kidney disease stage ≥3. Interviews will be informed by the Theoretical Framework of Acceptability.^18^ Interviews will be contextualised around the patient’s recent experiences of heart failure care, such as monitoring visits, treatment decision making, medicine-taking, information and support needs, and communication with healthcare professionals. Data analysis will initially be inductive, drawing on reflexive thematic approaches,^11^ then deductive, using the Theoretical Framework of Acceptability^18^ and informed by the HIAT.^17^

### Data oversight

The Chief Investigator will preserve the confidentiality of the participants taking part in the study and will abide by the Data Protection Act 2018 and the UK GDPR. The University of Liverpool is the data controller.

## Discussion

### Summary

Individualised identification of patients most at risk of declining renal function through monitoring, enabled by the algorithm and a clear standardised care pathway, should improve patient care and outcomes.^4^ Stages 1–6 of WP2, described in this protocol article, will provide strong evidence from primary care practices, practitioners, and patients by which to assess and refine the pathway, before it is tested in a cluster RCT in WP3.

### Strengths and limitations

Strengths include the use of behaviour change models, such as the COM-B and TDF in the design of research tools, the use of mixed quantitative and qualitative methods, and embedded PPIE across the whole research programme.

Anticipated challenges include delivering a complex research programme within the timescales available and managing collaborative partnerships across sectors, including contractual delays with external providers adapting electronic health records and providing access to clinical data.

### Implications for research and practice

This WP will co-design a new primary care intervention using behaviour change theory to incorporate an algorithm-guided individualised treatment pathway to maintain the renal function of patients treated for heart failure. This new pathway will then be tested in a cluster RCT.

## Data

The research is registered with the ISRCTN (reference: ISRCTN 75284771). Datasets will be uploaded into ResearchFish.
